# Leading the charge

**DOI:** 10.7554/eLife.37910

**Published:** 2018-06-07

**Authors:** Andrew JR Plested

**Affiliations:** 1 Institute of BiologyHumboldt Universität zu BerlinBerlinGermany; 2 Leibniz Forschungsinstitut für Molekulare Pharmakologie (FMP)BerlinGermany; 3 NeuroCureCharité UniversitätsmedizinBerlinGermany

**Keywords:** human serotonin transporter, surface charge, membrane capacitance, electrophysiology, cocaine, serotonin, Human

## Abstract

A simple label-free method uses the electrical properties of cells to detect how ligands bind to membrane proteins.

**Related research article** Burtscher V, Hotka M, Li Y, Freissmuth M, Sandtner W. 2018. A label-free approach to detect ligand binding to cell surface proteins in real time. *eLife* **7**:e34944. doi: 10.7554/eLife.34944

Membranes are thin slivers of fat that envelop cells, but on closer examination it becomes clear that the phospholipid bilayer that forms the membrane is more like a kind of sandwich. The water-repellent ‘tails’ of the phospholipids point inwards and form a core that acts as a barrier around the cell, whereas the ‘head’ groups (which are polar) orient outwards and attract water, ions and other charged molecules (Figure 1). Not so much keeping your friends close, as keeping your enemies closer.

**Figure 1.**
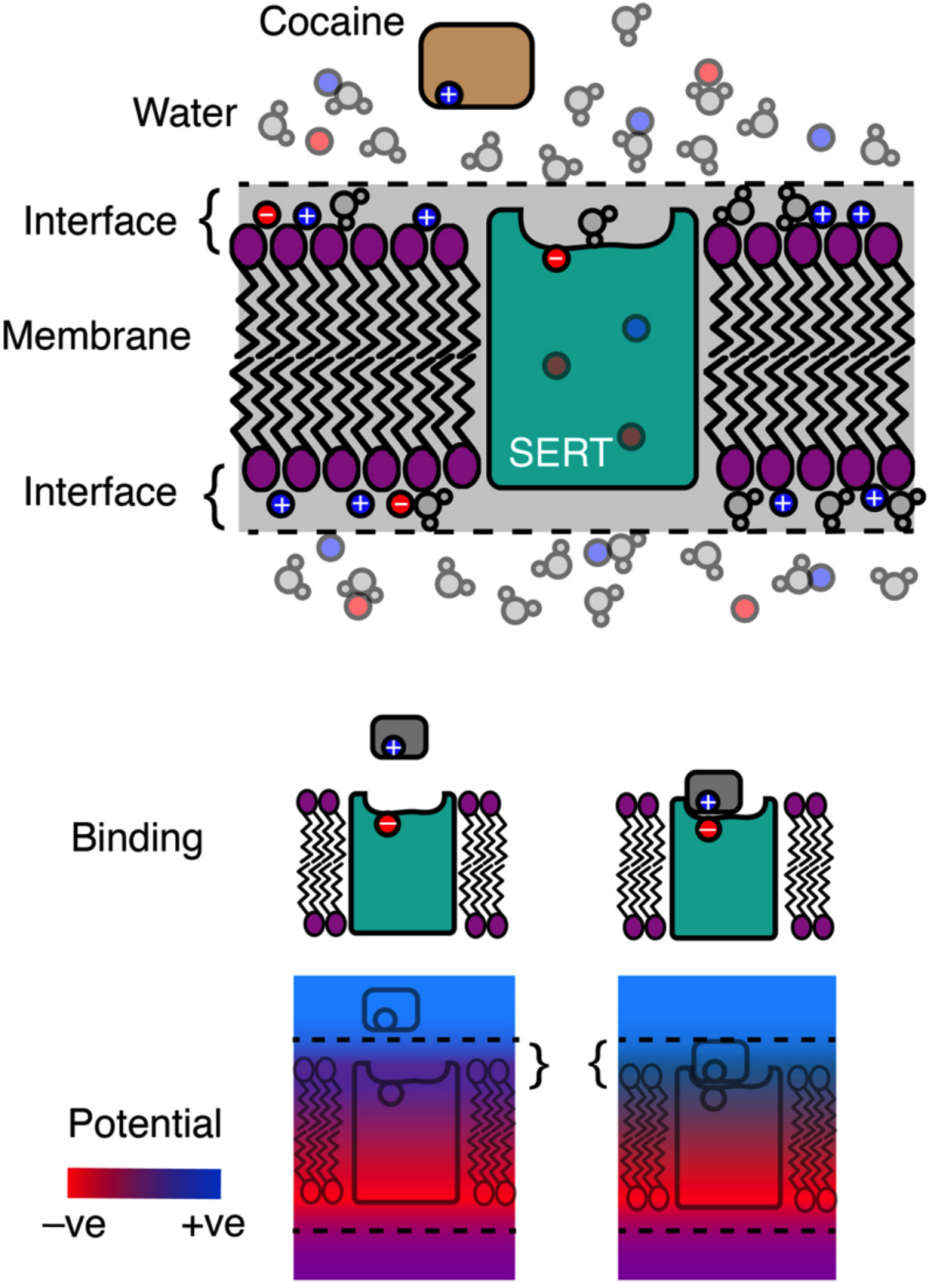
Label-free detection of ligand binding. Top: Cell membranes consist of a phospholipid bilayer in which the phospholipid ‘tails’ (zigzags) point inwards, while the polar ‘heads’ (purple ovals) form the membrane surface. On both sides of the bilayer there are interface regions that contain positive ions (blue), negative ions (red) and various polar molecules (including water) that have bound to the membrane. Proteins in the membrane, such as the serotonin transporter (SERT; cyan), allow charged molecules to cross it. These proteins also have a charge associated with their surface: in the case of SERT, a negative charge. This allows SERT to attract positively charged ligands such as cocaine (middle panel). Bottom: the binding of a positive ligand to SERT masks its exposed negative charge. The masking changes the potential (red, negative; blue, positive) in the interface region, making it more positive (as can be seen by comparing the areas marked with curly braces). Burtscher et al. show that this change in potential can be detected as a transient current or a long-lasting apparent capacitance change.

Because the membrane is so good at preventing charged objects from crossing it, cells employ numerous membrane proteins – pumps, secondary transporters and ion channels – to transport ions and all kinds of charged molecules. This is essential for a number of processes, including cell signalling, nutrient absorption, and muscle contraction. Anything that is charged needs to bind to the membrane proteins to be transported, so these proteins carry charged and polar groups to attract the particles. These groups, together with the head groups, contribute to the charged layer associated with the membrane surface.

The electrical properties of cell membranes have been actively studied for at least a century. Membranes have an electrical potential (usually measured in millivolts) across them that results from the unequal ion distributions inside and outside the cell; the action potential by which neurons conduct signals along their length is perhaps the most celebrated example of electrophysiology (Cole and Curtis, 1939).

Molecular scale information can be extracted by measuring the flow of charge in the cell membrane. Esoteric examples include brief ‘gating currents’ that accompany the activation of voltage-gated ion channels (Armstrong and Bezanilla, 1973), and brief current spikes that result from shape changes to the G-protein coupled receptors (Ben-Chaim et al., 2006). All this is just another way of saying that a few electrical charges per molecule matter a lot. Now, in eLife, Walter Sandtner and co-workers at the Medical University of Vienna – Verena Burtscher, Matej Hotka, Yang Li and Michael Freissmuth – report how apparent changes in the capacitance of the membrane can be used to measure the binding of charged ligands to membrane transporters (Burtscher et al., 2018).

Figure 1 illustrates the general principle of the measurement. Serotonin transporters (SERT) expose negative charges at the outer surface of the membrane. If a positively charged ligand like cocaine binds to the transporter, the net charge in the membrane (which is negative) is reduced. Burtscher et al. found that this change could be detected in whole cell patch clamp experiments.

Applying a square wave voltage to the membrane allows the charging and discharging of the cell membrane to be measured by studying the current that flows in response. The capacitance of the membrane (that is, its ability to store charge) can be calculated from this current and the potential of the membrane. Burtscher et al. could reliably and robustly detect apparent changes in capacitance that were associated with drug molecules binding to the transporters. Brief spikes in current also appeared as the drug first bound to the transporter; however, the capacitance change was a much clearer indicator of the binding.

Because there are many possible artefacts in the recorded current, Burtscher et al. went to considerable lengths to ensure that their results matched the predictions of prevailing theories for the electrochemistry of the charged layer. Indeed, the measurements did correspond with the Gouy-Chapman theory, which states that capacitance depends on the applied potential and the concentration of ions in solution. By buttressing their measurements with a solid theoretical approach, Burtscher et al. have developed a powerful method that could be used to detect processes other than ligand binding. For example, conformational changes that expose or cloak the charges on the surface of membrane proteins might be detected by a separate, slowly developing capacitance change.

The pattern of apparent capacitance reduction could also be used to determine the location of the sites on the transporters to which molecules bind. In what initially seemed to be a rather puzzling result, sufficiently high concentrations of the antidepressant desipramine (which, like cocaine, is positively charged and can bind to SERT) can alter the apparent capacitance of control cells that do not express SERT. Burtscher et al. determined that this confounding effect is because desipramine accumulates in cells and binds to the inner face of the membrane; the effect is absent in more acidic conditions when less of the uncharged form of desipramine is available to freely move across the membrane.

Inverting this logic suggests that a SERT inhibitor called ibogaine does not bind to the transporter at a site inside the cell, as had previously been thought. Ibogaine reduces the apparent capacitance of cells that express SERT, but has no effect control cells lacking SERT. This led Burtscher et al. to conclude that ibogaine binds to SERT at a site on the outer face of the cell.

The principal advantages of detecting ligand binding from capacitance changes are that conventional equipment and analysis can be used, and that molecules do not have to be labelled. What is perhaps surprising is that the changes at the surface of a single cell are reproducible enough to be quite easily resolved.

More work will be required to show how generally this method can be applied. It seems likely that the electrochemistry of individual ligands has an important effect on how they affect the apparent capacitance of the membrane they bind to. The approach is not obviously high-throughput but could in principle be adapted to a multiplex approach similarly to other patch clamp experiments, like the HERG screening that checks that new drugs won’t affect heart rhythms (Kiss et al., 2003). However, because so many membrane proteins deal with charged ligands, this capacitance method should be applicable to many situations and is therefore a welcome addition to the toolkit of membrane physiologists.
